# Estimation of genetic parameters for heifer pregnancy and carcass traits in angus cattle

**DOI:** 10.1093/jas/skag139

**Published:** 2026-05-09

**Authors:** Sarah L Phelps, Miranda M Culbertson

**Affiliations:** Department of Animal Science, Iowa State University, Ames, IA 50011, United States; Department of Animal Science, Iowa State University, Ames, IA 50011, United States

**Keywords:** beef cattle, carcass traits, heifer pregnancy

## Abstract

The genetic relationship between heifer pregnancy (HP) and carcass traits in purebred Angus cattle was evaluated in this study to expand industry knowledge of the impact of carcass trait selection on HP and the potential use of carcass traits as indicators of HP. Historical data consisting of 1,462 records for HP and 4,519 records for carcass traits from the Iowa State University McNay Memorial Research and Demonstration Farm were used in this study. A pedigree consisting of 10,455 individuals with parent generations ranging from 1 to 9 for sires and 1 to 11 for dams was used to build relationships among individuals. Genetic correlations between HP and carcass traits were analyzed using multivariate models consisting of HP, weaning weight (WWT), and a single carcass trait. Results from this study estimated a low heritability of 0.09 ± 0.06 for HP and estimates of 0.36 ± 0.04 for ribeye area (REA), 0.39 ± 0.04 for backfat thickness (FAT), 0.48 ± 0.04 for marbling score (MS), 0.38 ± 0.04 for hot carcass weight (HCW), 0.18 ± 0.03 for weaning weight direct (WWT_d_), and 0.07 ± 0.02 for weaning weight maternal (WWT_m_). Genetic correlations between HP and both carcass and weaning traits were negative with estimates of −0.32 ± 0.23 for REA, −0.22 ± 0.23 for FAT, −0.10 ± 0.22 for MS, −0.44 ± 0.21 for HCW, and −0.20 ± 0.25 for WWT_d_. All estimates of genetic correlation were accompanied by large standard errors. The results from this study suggest a potential antagonistic genetic relationship between heifer pregnancy and carcass trait performance in this herd of Angus cattle.

## Teaser

Genetic correlations between carcass traits and heifer pregnancy were negative, suggesting that selection for increased carcass trait performance could negatively impact heifer reproductive performance.

## Introduction

Heifer reproductive performance is an economically relevant trait in beef cattle production. Identifying the probability of a heifer becoming pregnant can aid in determining potential reproductive performance and replacement heifer selection. Conventionally, heifer pregnancy (HP) is measured as a binary trait of whether or not a yearling female became pregnant in her first breeding season. Genetic prediction of HP is traditionally evaluated using a threshold model, as it yields better response to selection and does not violate regression assumptions of linear models (Buddenberg et al. 1989; Misztal et al. 1989). However, recent literature has evaluated the use of alternative methods to evaluate HP predictions, such as random regression and evaluating HP on the observed scale, then transforming results to the probability scale (Giess 2024; Hidalgo et al. 2024).

Low to moderate heritability estimates that range from 0.12 to 0.24 have been generated through traditional HP models, indicating that this trait can be improved slowly through genetic selection (Doyle et al. 2000; Bormann et al. 2006; McAllister et al. 2011; Naya et al. 2017; Boldt et al. 2018; Giess 2024). For traits with low heritability, it may be advantageous to identify a second trait that is moderately to highly genetically correlated with the trait of interest and apply selection pressure on the second trait. Indirect selection is beneficial when the product of the genetic correlation between the trait of interest and a correlated trait, and the square roots of the respective heritabilities is greater than the heritability of the trait of interest. Carcass traits, such as ribeye area (REA), backfat thickness (FAT), marbling score (MS), and hot carcass weight (HCW), are moderate to highly heritable, and the American Angus Association reports estimates of 0.42 for REA, 0.39 for FAT, 0.48 for MS, and 0.40 for HCW (American Angus Association 2025). Furthermore, previous literature has suggested that genetic relationships may exist between HP and carcass traits; however, research on this topic is limited.

In the limited research available, it has been found that non-cyclic heifers have a lower percentage of intramuscular fat (IMF) and FAT, measured by ultrasound, compared to cycling heifers at the time of breeding (Evans et al. 2004). Several studies have identified FAT as influential for female fertility (Wettemann et al. 2003; Zieba et al. 2005). One study conducted in Australia compared heifers selected for either high or low FAT breeding values and found that heifers with higher FAT breeding values had increased pregnancy rates compared to those with low FAT (Jones et al. 2017). Genetic correlations between heifer fertility and carcass traits have yielded mixed results. In Red Angus cattle, genetic correlations with HP have been estimated as 0.21 ± 0.21 for REA, −0.08 ± 23 for FAT, −0.08 ± 19 for MS, and −0.03 ± 0.25 for HCW (Boldt et al. 2018). Other literature in Red Angus cattle has reported a 0.10 ± 0.15 genetic correlation between HP and MS using a sire model (McAllister et al. 2011). Genetic correlations between ultrasound carcass traits and HP have produced more promising results, with low positive estimated correlations of 0.16 ± 0.08 for ultrasound REA, 0.14 ± 0.08 for ultrasound FAT, and 0.06 ± 0.08 for ultrasound IMF (Boldt et al. 2018).

To date, genetic correlations between HP and carcass traits in Angus cattle have not been published. This presents an opportunity to further investigate the genetic relationship between HP and carcass traits in Angus cattle. The objective of this study was to explore the genetic relationship between HP and REA, FAT, and MS from a herd of registered Angus cattle that has placed selection pressure on marbling. This offers the potential to investigate the implications of carcass trait selection on reproductive performance and improve replacement heifer selection.

## Materials and methods

### Animal use

This study utilized historical data for evaluations, and approval from the Institutional Animal Care and Use Committee was not required.

### Data collection

Historical records for HP and carcass records were collected from the Iowa State McNay Memorial Research and Demonstration Farm between the years 1996 and 2024. An initial pedigree of 10,455 individuals was constructed to establish relationships among relatives. This pedigree included 162 unique sires and 902 unique dams, with sire generations ranging from 1 to 9 and dam generations from 1 to 11.

Pregnancy status was evaluated as a binary phenotype. Observations were coded as 1 if a heifer became pregnant and 0 if she failed to conceive within the breeding season, which lasted a maximum of 60 d. Pregnancy status was determined by evaluating breeding and calving records. Only records of heifers who were exposed for breeding, either artificial insemination or bull, received a phenotype record for HP. A heifer received a successful observation for pregnant (1) when she was exposed for breeding and calved the following year. If a heifer was included in exposure records but had no calving record, she was reported as not pregnant (0). Contemporary group (CG) formation for HP is provided in Table 1 and was defined by birth year and season. All heifers born within the same year and season would be managed together post-weaning and exposed for breeding at the same time; therefore, the yearling CG and exposure date was intrinsically accounted for. Data for HP consisted of 1,462 records and 18 CGs. The overall average pregnancy rate of the herd using data since 1996 was 72%.

**Table 1:** Definition of contemporary group (CG) and summary statistics to evaluate heifer pregnancy (HP), carcass traits, and weaning weight.

Trait	Distinct CG	Average size	Definition of CGs
**HP**	18	82 ± 32	Birth year, season
**Carcass**	94	48 ± 18	Birth year, season, management code, sex*, slaughter date
**Weaning**	263	29 ± 20	Birth year, season, management code, sex*, weaning date

* **Sex split by heifer, castrated male, and intact male.**

Carcass traits measured at slaughter included REA, FAT measured at the 12th rib, MS, and HCW. All MS were standardized using numerical scores for United States Department of Agriculture (USDA) Quality Grades and the corresponding MS, which were measured on a scale of 1.0 to 9.9 (BIF Guidelines Wiki 2021). Carcass trait CGs were defined by birth year and season, kill date, and sex, and are shown in Table 1. Kill date accounted for differences between slaughter plants, as no two groups were killed at separate locations on the same date. Three levels of sex were female, castrated male, and intact male. The carcass dataset included 4,517 records for REA, 4,518 for FAT, and 4,519 for MS from 223 unique sires and 1,629 unique dams. Across all carcass traits, 94 CGs were formed with an average size of 48 animals.

Weaning weight (WWT) information was also included in the multivariate models to account for selection bias of carcass traits and was partitioned into a direct, maternal, and maternal permanent environmental effect. Weight records on calves were collected at weaning, and a total of 7,648 animals across 285 CGs were used in this study, representing offspring from 240 unique sires and 1,923 unique dams. WWT CGs had an average size of 29 animals and were defined by birth year, season, management code, sex, and weaning date. Management code was used to account for differences in the management of cattle that were born in the same year and season and weaned at the same time.

### Statistical analysis

Estimates for variance components and genetic correlations were generated using the ASREML 4.2 software package (Gilmour et al. 2016). HP was evaluated using a probit link function, where the residual variance was fixed to one, to evaluate the binary response on the underlying scale (Gianola and Foulley 1983; Harville and Mee 1984). Four multivariate models were constructed to evaluate the relationships between HP and carcass traits. Each model included HP, WWT, and one carcass trait.

The multivariate models followed the general matrix form:


$$\left[\begin{array}{c} \begin{array}{c} y_{1}\\ y_{2} \end{array}\\ y_{3} \end{array}]=[\begin{array}{ccc} X_{1} & 0 & 0\\ 0 & X_{2} & 0\\ 0 & 0 & X_{3} \end{array}][\begin{array}{c} b_{1}\\ b_{2}\\ b_{3} \end{array}]+[\begin{array}{ccc} Z_{d1} & 0 & 0\\ 0 & Z_{d2} & 0\\ 0 & 0 & Z_{d3} \end{array}][\begin{array}{c} u_{d1}\\ u_{d2}\\ u_{d3} \end{array}]+[\begin{array}{ccc} 0 & 0 & 0\\ 0 & 0 & 0\\ 0 & 0 & Z_{m3} \end{array}][\begin{array}{c} 0\\ 0\\ u_{m3} \end{array}]+[\begin{array}{ccc} 0 & 0 & 0\\ 0 & 0 & 0\\ 0 & 0 & Z_{pe3} \end{array}][\begin{array}{c} 0\\ 0\\ u_{pe3} \end{array}]+[\begin{array}{c} e_{1}\\ e_{2}\\ e_{3} \end{array}\right]$$

Where $y_{i}$ was the vector of observations for the *i^th^* trait (1 = HP, 2 = a carcass trait, 3 = weaning weight), $b_{i}$ was the vector of unknown fixed effect solutions, ***u_di_*** was the vector of random direct additive genetic effects, ***u_mi_*** was the vector of maternal additive genetic effects, ***u_pei_*** was the vector of maternal permanent environment effects, ***e_i_*** was a vector residual effects, and ***X_i_, Z_di_, Z_mi_, Z_pei_*,** were incidence matrices corresponding to $b_{i}$**, *u_di_, u_mi_*,** and***u_pei_***, respectively.

The variances and means of the model were as follows:

$E(y_{1})=\Phi \;(\beta_{0}+\;X_{1}b_{1})\;$ for HP;

$E(y_{i})=X_{i}b_{i}$ for carcass traits and weaning weight


$$E(e_{i})=E(u_{i})=0$$

$$\mathrm{var}[\begin{array}{c} u_{d1}\\ u_{d2}\\ u_{d3}\\ u_{m3}\\ u_{pe3}\\ e_{1}\\ e_{2}\\ e_{3} \end{array}]$$

$$=[\begin{array}{c} \begin{array}{c} \begin{array}{cccccccc} {A\sigma^{2}}_{d1} & A\sigma_{d1,d2} & A\sigma_{d1,d3} & A\sigma_{d1,m3} & 0 & 0 & 0 & 0\\ A\sigma_{d1,d2} & {A\sigma^{2}}_{d2} & A\sigma_{d2,d3} & A\sigma_{d2,m3} & 0 & 0 & 0 & 0\\ A\sigma_{d1,d3} & A\sigma_{d2,d3} & {A\sigma^{2}}_{d3} & A\sigma_{d3,m3} & 0 & 0 & 0 & 0\\ A\sigma_{d1,m3} & A\sigma_{d2,m3} & A\sigma_{d3,m3} & {A\sigma^{2}}_{m3} & 0 & 0 & 0 & 0\\ 0 & 0 & 0 & 0 & I_{pe3}\sigma_{pe3}^{2} & 0 & 0 & 0\\ 0 & 0 & 0 & 0 & 0 & I_{e1}\sigma_{e1}^{2} & 0 & 0\\ 0 & 0 & 0 & 0 & 0 & 0 & I_{e2}\sigma_{e2}^{2} & 0\\ 0 & 0 & 0 & 0 & 0 & 0 & 0 & I_{e3}\sigma_{e3}^{2} \end{array} \end{array} \end{array}]$$

Where ***A*** was the numerator relationship matrix, ***I_pe3_*** was the identity matrix with an order of the number of dams for trait *i*, ***I_ei_*** was the identity matrix with an order equal to the number animals for the *i^th^* trait, $\sigma_{\mathrm{di}}^{2}$ was the direct additive genetic variance for the trait *i*, σdi,di' was the direct genetic covariance between the traits, $\sigma_{\mathrm{di},\mathrm{mi}}$ was the direct maternal genetic covariance for the *i^th^* trait, $\sigma_{m}^{2}$ was the maternal additive genetic variance for WWT, $\sigma_{\mathrm{pe}}^{2}$ was the maternal permanent environmental variance for WWT, and $\sigma_{\mathrm{ei}}^{2}$ was the residual variance between the traits and was fixed to one for HP, and Φ denotes the probit transformation that is a function of the standard normal cumulative distribution.

## Results

Summary statistics for HP and carcass traits are reported in Table 2. Across all years, the average HP rate was 72%. The average carcass and weaning measurements were 80.92 ± 8.17 cm^2^ for REA, 12.14 ± 4.44 mm for FAT, 5.50 ± 1.47 for MS, 335.10 ± 194.59 kg for HCW, and 195.28 ± 44.69 kg for WWT.

**Table 2:** Summary statistics for heifer pregnancy, carcass traits, and weaning weight from historical phenotypic records collected from 1998 to 2023 on Angus cattle.

Trait^1^	n^2^	Min^2^	Max^2^	Mean	SD^2^
**^3^HP**	1462	0.00	1.00	0.72	0.45
**REA, cm²**	4517	45.16	118.06	80.92	8.17
**FAT, mm**	4518	1.78	48.26	12.14	4.44
**MS**	4519	1.00	9.89	5.50	1.47
**HCW, kg**	4520	194.60	508.93	335.10	194.59
**WWT, kg**	7638	49.5	360	195.28	44.69

1 HP = heifer pregnancy, REA = ribeye area, FAT = backfat thickness, MS = marbling score, HCW = hot carcass weight, WWT = weaning weight.

2 n= number of animals, Min = minimum, max = maximum, SD = standard deviation.

3 HP was evaluated as a binary response (1 = pregnant, 0 = not pregnant).

For modeling, the residual variance for HP was fixed to one in all models due to the nature of probit link models. A limited number of 86 heifers had both HP and carcass records, and all failed to conceive and were subsequently slaughtered. Therefore, the residual covariance between HP and carcass traits were restricted to zero as records on both traits are needed to estimate environmental influences. The genetic covariance between WWT_m_ and all traits except WWT_d_ was fixed to zero.

Variance component and heritability estimates from multivariate models are reported in Table 3. Since all models included HP and WWT, the reported estimates represent the mean across models, and the standard error reflects the largest observed. The heritability estimate for HP was 0.09 ± 0.06, indicating that it is a lowly heritable trait. Heritability of carcass traits were moderate to high, with estimates of 0.36 ± 0.04 for REA, 0.39 ± 0.04 for FAT, 0.48 ± 0.04 for MS and 0.38 ± 0.04 for HCW. WWT traits were lowly heritable with estimates of 0.18 ± 0.03 for WWT_d_ and 0.07 ± 0.02 for WWT_m_. The maternal permanent environmental effect of the damn on WWT was estimated as 0.15 ± 0.02.

**Table 3:** Multivariate model estimates for additive genetic variance ($\sigma_{a}^{2}$), phenotypic variance ($\sigma_{p}^{2}$), heritability (h²), and maternal permanent environmental effect (C²) for heifer pregnancy (HP), carcass, and weaning weight (WWT) traits.

Trait^1^	$\sigma_{a}^{2}$	$\sigma_{p}^{2}$	h²	C²
**HP**	0.10 ± 0.06	1.10 ± 0.06	0.09 ± 0.06	
**REA**	17.59 ± 2.31	49.31 ± 1.25	0.36 ± 0.04	
**FAT**	4.02 ± 0.52	10.26 ± 0.27	0.39 ± 0.04	
**MS**	0.46 ± 0.05	0.96 ± 0.03	0.48 ± 0.04	
**HCW**	403.21 ± 45.77	1045.60 ± 26.31	0.38 ± 0.04	
**WWT_d_**	117.31 ± 23.93	646.20 ± 19.30	0.18 ± 0.03	
**WWT_m_**	47.97 ± 16.29	646.20 ± 19.30	0.07 ± 0.02	
**WWT_pe_**				0.15 ± 0.02

1 HP = heifer pregnancy, REA = ribeye area, FAT = backfat thickness, MS = marbling score, HCW = hot carcass weight, WWT_d_ = weaning weight direct, WWT_m_ = weaning weight maternal, WWT_pe_ = permanent maternal effect of the dam on weaning weight.

Estimates of genetic covariance and correlations between HP and carcass traits and WWT are shown in Table 4. All genetic correlations between HP and the other traits were negative, suggesting a potential antagonistic relationship. The genetic correlations between HP and all traits were as follows: −0.32 ± 0.23 for REA, −0.22 ± 0.23 for FAT, −0.10 ± 0.22 for MS, −0.44 ± 0.21 for HCW, and −0.20 ± 0.25 for WWT_d_. No genetic correlations were estimated between HP and WWT_m_ as the genetic covariance was constrained to zero. Furthermore, since the residual variance for HP was restricted to 1, residual covariances were not estimable.

**Table 4:** Estimates of genetic covariances (COV_a_) and genetic correlations (r_a_) between heifer pregnancy (HP) and carcass traits and weaning weight.

Trait^1^	COV_a_	r_a_
**REA**	−0.43 ± 0.31	−0.32 ± 0.23
**FAT**	−0.14 ± 0.15	−0.22 ± 0.23
**MS**	−0.02 ± 0.05	−0.10 ± 0.22
**HCW**	−2.96 ± 1.38	−0.44 ± 0.21
**WWT_d_**	−0.70 ± 0.86	−0.20 ± 0.25

1 HP, heifer pregnancy; REA, ribeye area; FAT, backfat thickness; MS, marbling score; HCW, hot carcass weight; WWT_d_, weaning weight direct.

## Discussion

Previous studies have estimated the heritability of HP to be low, with estimates from threshold models to be between 0.12 and 0.24 (Doyle et al. 2000; Bormann et al. 2006; McAllister et al. 2011; Naya et al. 2017; Boldt et al. 2018; Giess 2024). In this study, the estimated heritability of HP was slightly lower but similar to previously observed estimates. Since this study utilized data from a single herd, total genetic variance for this trait could be lower than what would be observed across a breed population and could explain the lower heritability. Furthermore, breeding heifers are preselected prior to measuring HP. Therefore, selection bias is an issue with estimating HP variance components.

The heritability of HP estimated from this study is comparable to other estimates reported in industry and previous studies. The American Angus Association reports the heritability of REA, FAT, MS, HCW, WWT_d_, and WWT_m_ as 0.42, 0.39, 0.48, 0.40, 0.28, and 0.12, respectively (American Angus Association 2025).

The current study estimated the heritability of these traits to be of 0.36, 0.39, 0.48, 0.38, and 0.18 for REA, FAT, MS, HCW, and WWT_d_, respectively. In Angus cattle, previous literature estimated a range of heritability for carcass traits, with a range of 0.32–0.59 for REA, 0.16–0.35 for FAT, 0.26–0.61 MS, and 0.29–0.73 for HCW (Wilson et al. 1993; Morris et al. 2000; Minick et al. 2004; MacNeil et al. 2010; Mao et al. 2013; Torres-Vázquez et al. 2018). Additional research performed with breeds of cattle other than Angus has reported similar heritability estimates for REA and MS, but slightly lower heritability of FAT (Boldt et al. 2018; Weik et al. 2021). In Red Angus cattle, the heritability of 0.35 has been reported for MS (McAllister et al. 2011).

It was determined that restricting the residual covariance to zero between carcass traits and WWT_m_ was best for this dataset. Previous research has estimated low to moderate genetic correlations between WWT_m_ and carcass traits, suggesting that the maternal genetic influence is still prominent post-weaning upon carcass trait measurements (Splan et al. 2002). However, the maternal influence has been shown to have lesser importance on beef cattle carcass traits compared to direct genetic effects (DeRouen et al. 1992). A previous study found the direct effect of traits measured at weaning was inflated if maternal effects were not accounted; however, the maternal influence diminished post-weaning and had no influence on final weight (Meyer 1992).

Low to moderate negative correlations were estimated between HP and all traits from multivariate models. These estimates slightly differ when compared to results from previous studies; however, results from previous studies have been inconsistent. A similar study in Red Angus cattle estimated genetic correlations with HP to be 0.21 ± 0.21 with REA, −0.08 ± 0.23 with FAT, −0.08 ± 0.19 with MS, and −0.03 ± 0.25 with HCW (Boldt et al. 2018). Negative correlations between HP and both FAT and REA are consistent with the findings from this study, but positive correlations between HP to REA and HCW differ. In both the current study and the study performed by Boldt et al. (2018), all genetic correlations between HP and carcass traits were followed by large standard errors. Another similar study estimated genetic correlations between reproductive traits measured on heifers and carcass traits measured in steer half-siblings (Splan et al. 1998). Estimates of genetic correlations between calving rate (evaluated identical to HP) and carcass traits were presented as 0.15 with REA, 0.19 with FAT adjusted for distortion, -0.05 with MS, and 0.05 with HCW. Results for correlations between HP and MS are similar to the current study, while all other carcass correlations differ in direction.

Estimating genetic correlations between carcass traits and fertility traits relies heavily on pedigree information, as it is uncommon for both traits to be measured on the same animal. In the current study, 86 animals had records on both HP and carcass traits, but all HP phenotypes were zero, indicating these females did not get bred and were sent to slaughter. To collect phenotypic information regarding both reproductive and carcass traits on the same animal, previous studies have used carcass ultrasound technology. Early research noted that non-cyclic heifers had a lower percentage of ultrasound IMF compared to cyclic heifers (Evans et al. 2004). The genetic correlation between HP and carcass MS was estimated as 0.10 using a sire model (McAllister et al. 2011). Using ultrasound values, genetic correlations between HP and carcass ultrasound traits were estimated to be low and positive with estimates of 0.16, 0.14, and 0.06 for REA, FAT, and IMF, respectively (Boldt et al. 2018). Additional research between carcass ultrasound traits and other heifer fertility traits yielded similar results. In tropical composite cattle, genetic correlations between days to first calving and ultrasound measurements for REA, FAT, and IMF were reported as −0.18, 0.21, and −0.30, respectively, suggesting that heifers with larger REA and more IMF conceived earlier compared to heifers with more FAT. (Facy et al. 2023). In Nelore cattle, estimated negative genetic correlations between age at first calving and both REA and FAT with estimates of −0.25 and −0.35, respectively (Caetano et al. 2013).

Results for WWT also differed between the current study and the Red Angus study, as the genetic correlation between HP and WWT direct was 0.18 (Boldt et al. 2018). In the current study, a low, negative genetic correlation was estimated between HP and WWT_d_. This suggests that an increased direct genetic influence on WWT has antagonistic effects on pregnancy outcome. In Brangus cattle, a genetic correlation of −0.28 was reported between HP and WWT_d_ (Fortes et al. 2012). However, it is important to consider that Brangus cattle are later maturing compared to Bos taurus breeds, such as Red Angus and Angus. Related studies concerning genetic correlations between WWT and fertility traits suggest a favorable relationship between the two traits. A study estimating genetic correlations between growth traits and age at puberty estimated a correlation of 0.07 between WWT and HP (Morris et al. 2000). With rebreeding, genetic correlations to WWT have been estimated as −0.08 with WWT_d_ and 0.48 with WWT_m_ (Weik et al. 2021). Genetic correlations for age at first calving have been estimated as 0.04 with WWT in Hanwoo cattle, 0.10 in Nelore cattle, and −0.23 in Canchim cattle (Pereira et al. 2001; Pires et al. 2017; Lopez et al. 2020).

## Conclusion

The heritability of HP was estimated as 0.09 ± 0.06 in this study from multivariate models. This indicates that genetic progress can be made slowly through selection in this purebred Angus herd. For carcass traits and WWT, heritability estimates were moderate to high and positive, indicating a quicker response to selection for improvement of these traits. Carcass trait heritability estimates from this study were as follows: 0.36 ± 0.04 for REA, 0.39 ± 0.04 for FAT, 0.48 ± 0.04 for MS and 0.38 ± 0.04 for HCW. WWT heritability estimates were 0.18 ± 0.03 for WWT_d_ and 0.07 ± 0.02 for WWT_m_, and the maternal permanent environmental effect of the damn on WWT was estimated as 0.15 ± 0.02.

Negative genetic correlations were estimates between all traits and HP, with estimates of −0.32 ± 0.23 for REA, −0.22 ± 0.23 for FAT, −0.10 ± 0.22 for MS, −0.44 ± 0.21 for HCW, and −0.20 ± 0.25 for WWT_d_. These findings suggest there could be an antagonistic relationship between carcass traits measured on feedlot animals and HP outcome measured on breeding heifers in the Iowa State University McNay Angus herd. However, estimates for genetic correlations in this study are accompanied by large standard errors due to minimal phenotypic measurements of both fertility and carcass traits on the same animals. Previous work utilizing carcass ultrasound traits in Red Angus cattle has reported low positive genetic relationships with heifer fertility traits, with more precise estimates. Therefore, alternative methods such as carcass ultrasound technology may provide better insight into the relationship between carcass traits and HP.

## Data Availability

The data underlying this article will be shared on reasonable request to the corresponding author.

## Abbreviations

CG: contemporary group
FAT: backfat thickness
HCW: hot carcass weight
HP: heifer pregnancy
IMF: intramuscular fat
MS: marbling score
REA: ribeye area
WWT: weaning weight
WWT_d_: weaning weight direct
WWT_m_: weaning weight maternal
